# Chemical Composition and Antioxidant Activity of *Ammi visnaga* L. Essential Oil

**DOI:** 10.3390/antiox11020347

**Published:** 2022-02-10

**Authors:** Fatima Zahra Kamal, Gabriela Dumitrita Stanciu, Radu Lefter, Valeriu V. Cotea, Marius Niculaua, Daniela Carmen Ababei, Alin Ciobica, Abdellah Ech-Chahad

**Affiliations:** 1 Laboratory of Physical Chemistry of Processes and Materials, Faculty of Sciences and Techniques, Hassan First University, B.P. 539, Settat 26000, Morocco; fatimzahra.kamal@gmail.com (F.Z.K.); echchahad@gmail.com (A.E.-C.); 2 Laboratory of Agri-Food and Health, Faculty of Sciences and Techniques, Hassan First University, B.P. 539, Settat 26000, Morocco; 3 Advanced Research and Development Center for Experimental Medicine (CEMEX), Grigore T. Popa University of Medicine and Pharmacy, 16 Universitatii Street, 700115 Iași, Romania; 4 Biomedical Research Center, Romanian Academy, Iași Branch, 8th Carol I Avenue, 700506 Iași, Romania; radu_lefter@yahoo.com; 5 Department of Oenology, "Ion Ionescu de la Brad" University of Life Sciences, 3rd M. Sadoveanu Alley, 700490 Iași, Romania; vcotea@uaiasi.ro; 6 Research Centre for Oenology Iași, Romanian Academy, Iași Branch, 9th M. Sadoveanu Alley, 700505 Iași, Romania; niculaua@gmail.com; 7 Pharmacodynamics and Clinical Pharmacy Department, Grigore T. Popa University of Medicine and Pharmacy, 16 Universitatii Street, 700115 Iași, Romania; dana.ababei@gmail.com; 8 Department of Biology, Faculty of Biology, Alexandru Ioan Cuza University, 11th Carol I Avenue, 700506 Iași, Romania

**Keywords:** *Ammi visnaga* L., chemical composition, antioxidant activity, essential oil, gas chromatography-mass spectrometry, oxidative stress

## Abstract

The present study evaluated the chemical composition and the in vitro and in vivo antioxidant potential of *Ammi visnaga* L. essential oil to provide a scientific basis for the use of this plant in the traditional pharmacopoeia. Gas chromatography-mass spectrometry was used to identify the volatile constituents present of the oil. The in vitro antioxidant capacity was evaluated by the DPPH and the reducing power assays. For the in vivo tests, oral administration of *Ammi visnaga* L. oil (600 and 1200 mg/kg body weight) was performed in Swiss albino mice treated with acetaminophen (400 mg/kg). The toxic effect of acetaminophen and the action of the essential oil were measured by determining the levels of lipid peroxidation and antioxidant enzymes in liver and kidneys homogenates. The major components identified were butanoic acid, 2-methyl-, pentyl ester, (Z)-β-ocimene, D-limonene, linalool, pulegone and lavandulyl-butyrate. The in vitro DPPH and reducing power assays showed moderate to low free radical scavenging activity and the antioxidant power was positively correlated with the polyphenols’ concentration. In vivo, the *Ammi visnaga* L. essential oil showed a high antioxidant capacity at both concentrations (600 and 1200 mg/kg), effectively increasing the levels of reduced glutathione, superoxide dismutase, and catalase and significantly reducing the lipid peroxidation. The results obtained from this study suggest that *Ammi visnaga* L. could represent a source of molecules with antioxidant potential in the prevention of free radical-related diseases.

## 1. Introduction

The reactive oxygen species (ROS) have been shown to act as a backbone of the immune system and to form the key component of the phagocytic cells and apoptotic processes, when produced at moderate concentrations through endogenous processes by mitochondria and various intracellular enzymes (glucose oxidase, myeloperoxidase, phospholipase A, etc.) and exogenous sources, such as environmental agents (pollution, radiation, and UV), cigarette smoking, drugs, and certain food [1]. However, when the ROS production rate exceeds the disposal rate, this leads to oxidative stress, a harmful molecular state, responsible for the oxidation of proteins, lipids, and DNA [1]. The damage to the structure and functioning of these macro-molecules, and the misinterpretation of protein translation mechanisms and genetic information arising thereof, can end up in several human diseases, including coronary heart disease, Alzheimer’s disease, and aging [2,3,4].

To thwart the disease-mediator oxidative damage, the tissues possess endogenous antioxidant defense systems of non-enzymatic type (glutathione, bilirubin, and coenzyme Q10) and enzymatic type, such as superoxide dismutase (SOD), catalase (CAT), and glutathione peroxidase (GSH-Px) [5]. These molecules ensure the scission of the auto-oxidative chain reaction, the conversion of radical species to non-radical or less toxic species and diminish the localized oxygen concentrations [5]. However, under the pressure of a wide variety of cellular homeostasis disrupting factors, ranging from metabolic [6] and mental stress conditions [7], to lifestyle [8], dietary [9], and aging conditions [10,11], endogenous antioxidants may prove insufficient to neutralize ROS generation and, therefore, call for exogenous antioxidant intake (dietary) to maintain optimal cellular functions [9,12]. Among the natural antioxidants, secondary and primary metabolites present in aromatic and medicinal plants have been shown to have a powerful antioxidant potential [13].

The antioxidant capacity of the Apiaceae family (Umbelliferae) has been extensively studied, showing very promising potential [14,15,16]. *Ammi visnaga* L. (*Daucus visnaga* L., *Visnaga daucoides* Gaertn.), a member of the Apiaceae, commonly known as Toothpick weed in England, or Khella in Arab countries, has only recently been reviewed for its numerous curative properties, out of which the antioxidant ones have been approached by very few studies [17]. A Northern hemisphere widespread biennial or annual herbaceous plant [17,18], *A. visnaga* L. presents an erect, cylindrical, and highly branched stem reaching 130 cm in height, covered with greyish green foliage. Its white flowers are grouped into umbels of 6–10 cm in diameter and the fruits are tiny, ovoid, and smooth formed of two partial mericarps, each 2 mm long greyish brown in color when the plant is dry [17]. In the popular pharmacopoeia, the plant is used as dried powder or boiled in water to treat renal colic, mild angina symptoms, asthma, spastic bronchitis, abdominal cramps, urinary calculi, vitiligo and psoriasis, vertigo, diabetes, and kidney stones [18]. In the pharmaceutical industry, various prescription drugs, such as amiodarone (ventricular arrhythmias), nifedipine (treatment of stable, variant, and unstable angina, mild to severe hypertension, and Raynaud’s phenomenon), and cromolyn (mastocytosis) are derived from *A. visnaga* L. [18].

In the frame of the abovementioned literature data, the present study aimed to investigate/establish the chemical composition of the essential oil of *A. visnaga* L. (from northwest Morocco), testing (by the way for the first time to our knowledge) both its in vitro and in vivo antioxidant activity.

## 2. Materials and Methods

### 2.1. Chemical Characterization of A. visnaga Essential Oil

#### 2.1.1. Plant Material

Healthy samples of *A. visnaga* L., without signs of contamination (fungal, bacterial, or viral), were collected at dawn during the June–July months, 2021, at flowering stage in their natural habitat, from Ouazzane (North Morocco) (34°48′ North, 5°36′ West, Altitude 614 m). After taxonomic identification, a specimen was deposited in the herbarium for future reference (Voucher n° 0356/M) at the Faculty of Sciences and Technologies, Hassan 1st University, Settat, Morocco. The plant was carefully washed with sterile distilled water, to remove dust and foreign matter, then shade dried and ground to fine powder using an electric grinder.

#### 2.1.2. Isolation of Essential Oil

A quantity of 500 g of *A. visnaga* powder were placed in a flask with 4 L of distilled water, and then subjected to hydro distillation for 6 h using a Clevenger type apparatus (JP Selecta, Barcelona, Spain). The golden-yellow essential oils obtained were dried on sodium anhydrous sulphate, and then stored at +4 °C in amber glass bottles with screw caps (to avoid the negative effect of light until the test).

#### 2.1.3. Chemicals and Reagents

Ascorbic acid (AA), aluminum chloride (AlCl_3_), iron chloride (FeCl_3_), Folin-Ciocalteu reagent, 2,2′-Diphenyl-1-picrylhydrazyl (DPPH), nitro blue tetrazolium (NBT), reduced glutathione (GSH), superoxide dismutase (SOD), catalase (CAT) from bovine liver, 1,2-dithio-bis nitro benzoic acid (DTNB), thiobarbituric acid (TBA) and trichloroacetic acid (TCA), acetaminophen (APAP), catechin, quercetin, ethylenediaminetetraacetic acid (EDTA), gallic acid, vanillin, nicotinamide-adenine dinucleotide phosphate (NADPH), butylated hydroxytoluene, phenazine methosulfate, sodium carboxymethyl cellulose (CMC), and n-alkanes (C6-C30) were purchased from Sigma Co. (St. Louis, MO, USA). Potassium phosphate monobasic, sodium pyrophosphate dibasic, sodium carbonate (Na_2_CO_3_), sodium hydroxide (NaOH), sodium nitrite (NaNO_2_), disodium hydrogen phosphate (Na_2_HPO_4_), hydrogen peroxide (H_2_O_2_), potassium ferricyanide [K_3_Fe(CN)_6_], sodium sulphate anhydrous (Na_2_SO_4_), hydrochloric acid, acetic acid (ACA), and n-butanol (99.8%) were of analytical grade and purchased from Merck (Nottingham, UK).

#### 2.1.4. Gas Chromatography–Mass Spectrometry of Essential Oil

The gas chromatography–mass spectrometry (GC-MS) analysis was performed without derivatization using gas chromatograph (Agilent 7890A Series) coupled to a mass spectrometer (MS) (5975C) (Agilent Technologies, Santa Clara, CA, USA) equipped with a multimode injector and a 123-BD11 column (ASTM D6584) (Agilent Technologies, Santa Clara, CA, USA) with a dimension of 15 m × 320 μm × 0.1 μm at the Moroccan Foundation for Advanced Science, Innovation and Research (MAScIR) Institute. In total, 10 µL of the liquid samples were dissolved in an appropriate volume of chloroform. Then, 4 µL of the soluble extract was injected into the column by 1:5 split mode using helium as carrier gas at a flow rate of 2 mL min^−1^. The composition of the essential oil determined from the peak areas was calculated as a percentage of the total compounds existing in the sample detection using full scan mode between 30–1000 *m*/*z*, with a gain factor of 5 and electron impact ionization. The temperatures of the ion source and the quadrupoles were 230 and 150 °C, respectively. The oven temperature was programmed at 30 °C for 3 min and then increased by 10 °C min^−1^ to 250 °C. The compounds identification was carried out using the NIST 2017 MS Library (https://chemdata.nist.gov/dokuwiki/doku.php?id=chemdata:start (accessed on 25 January 2022)). A series of n-alkanes (C6–C30) were used in this experiment to calculate the Retention Index.

### 2.2. The In Vitro Antioxidant Assay

#### 2.2.1. Total Polyphenol Determination

The total phenolic content was determined by the colorimetric method of Folin–Ciocalteu described by Singleton and Rossi [19]. A measured 2 mL of sodium carbonate Na_2_CO_3_ (75 g/L) was added to the mixture of 0.5 mL of sample and 2.5 mL of 10% Folin-Ciocalteu reagent. The absorbance was measured at 760 nm, after 30 min of incubation at room temperature, and in the dark against the blank. The results were expressed as gallic acid equivalent per gram of extract (mg GAE/g). Three tests were performed for each sample.

#### 2.2.2. Total Flavonoid Determination

Total flavonoid content was determined by the method described by Zhishen et al. and Kim et al. [20,21]. A volume of 500 μL of oil (prepared in methanol) was mixed with 120 μL of 5% NaNO_2_. After 5 min, 120 μL of 10% AlCl_3_ (freshly prepared) was added; the medium was shaken to homogenize the contents. After 6 min of incubation at room temperature and in the dark, 800 μL of NaOH (1M) was added. The solution was thoroughly homogenized, and the absorbance was read immediately at 430 nm against a blank. The standard curve for total flavonoids was made using quercetin standard solution. The results were expressed as milligrams of quercetin equivalent per gram of extracts (mg Quercetin Equivalent (QE)/g).

#### 2.2.3. Condensed Tannins Determination

The content determination of condensed tannins (known as proanthocyanidins) was done by using the colorimetric method; this method is based on the depolymerization of these molecules in the presence of sulfuric acid, followed by the formation of anthocyanidols of a specific red color, in the presence of vanillin, analyzable at 500 nm. In a test tube, 1 mL aliquot of the extract is added to 3 mL of 4% vanillin and 1.5 mL of concentrated HCl. After 15 min of incubation at room temperature, the absorbance was measured at 500 nm. The tannin content was determined from the calibration curve, performed in parallel under the same operating conditions using catechin as positive control [22].

#### 2.2.4. 2,2′-Diphenyl-1-picrylhydrazyl (DPPH) Assay

The free radical scavenging activity of *A. visnaga* L. was measured using the DPPH assay. Quantitative estimation of radical scavenging activity was measured according to the protocol described by Bougandoura et al. [23]. In total, 2 mL of freshly prepared 0.1 mM methanolic DPPH was added to essential oil (0.5 mL; 0–1 mg/mL), and the mixture was shaken vigorously for 5 min using a vortex, and incubated 30 min in the dark, at room temperature. Absorbance was measured at 517 nm with a VR-2000 spectrophotometer (JP Selecta, Barcelona, Spain) against a blank, and compared with standard (butylated hydroxytoluene (BHT)). Scavenging activity was expressed as the percentage inhibition (I%) calculated by the following equation [24]:

I% = (A_blank_ − A_sample_/A_blank_) × 100

The IC_50_ was calculated graphically by linear regression of I% versus concentrations (C).

#### 2.2.5. Reducing Power Assay

The reducing power of *A. visnaga* essential oil was determined by the method of Oyaizu [25]. A volume of 1 mL of samples or standard antioxidants was mixed with phosphate buffer (2.5 mL, 0.2 M, pH 6.6) and 2.5 mL of 1% *w*/*v* potassium ferricyanide K_3_[Fe(CN)_6_]. After 20 min of incubation at 50 °C in the dark, 2.5 mL of 10% TCA was added to stop the reaction; the tubes were centrifuged at 3000 rpm for 10 min. Then, 2.5 mL of supernatant was mixed with 2.5 mL of distilled water and 0.5 mL of 0.1% aqueous FeCl_3_ solution. The absorbance was read at 700 nm against a blank and compared with standard (AA 0–1 mg/mL). The results were expressed as mg AA per g sample. All tests were performed in triplicates.

### 2.3. The In Vivo Antioxidant Assay

#### 2.3.1. Animal Models and Induction of Oxidative Stress

Thirty adult male Swiss albino mice, with an initial body weight of 25–35 g, were housed in polypropylene cages (5 mice/cage) containing wood shavings bedding, provided with a label holder mentioning the name of the batch, and placed in a controlled environment (T = 22 ± 3 °C, relative humidity 40–70%, 12 h light/dark cycles “lights on at 7:00 a.m.”, with standard mice chow and water ad libitum). For the induction of oxidative stress, mice received APAP (400 mg/kg) by intraperitoneal injection (ip) once, as an acute toxic dose.

#### 2.3.2. Study Design

The test consisted of measuring the effects of different doses of the essential oil of *A. visnaga* L. on the biochemical parameters of the tissue homogenates of mice GSH, CAT, SOD, and malondialdehyde (MDA). For this purpose, mice were randomly divided into six groups (*n* = 5):

- Group I was designated as vehicle and was treated with 0.1% CMC;
- Group II (negative control) received no treatment but had free access to water and food;
- Groups III (toxic control), IV, V, and VI received a single intraperitoneal injection of APAP (400 mg/kg, ip) before the start of the experiment to induce hepato-renal oxidative injury. Group IV served as the standard and received AA, 200 mg/kg body weight. Groups V and VI received *A. visnaga* L. essential oil at doses of 600 and 1200 mg/kg body weight.

These doses were selected following a screening procedure in which we tested three doses (600, 1200, and 1800 mg/kg) of *A. visnaga* L. essential oil by oral administration in rats for 2 weeks (unpublished results). At the highest concentration (1800 mg/kg), we observed installation of the LD50, whereas the other two doses did not provoke signs or symptoms of toxicity and were selected for further antioxidant studies. The animals were treated orally, once a day (at 9 a.m.) for two weeks (14 days). A 10-day quarantine was observed before treatment [26].

#### 2.3.3. Body and Organ Weight

The mice were weighed twice (at baseline to endpoint), and the changes in body weight were recorded. After the mice were sacrificed, the liver and kidneys were weighed accurately, and the relative weight was calculated as follows:

Relative organ weight = (absolute organ weight (g) × 100)/body weight of mice on sacrifice day (g).

#### 2.3.4. Preparation of Tissue Homogenates

After euthanasia of the mice, the liver and kidney samples were collected and rinsed immediately in ice-cold saline, dried, and weighed. Then, the samples were ground and homogenized in cold potassium phosphate (50 mM, pH 7.0, containing 1 mM EDTA) per gram of tissue. The homogenate was centrifuged at 10,000× *g* for 15 min at 4 °C, to purge cellular debris and the supernatants were collected [27]. The levels of MDA, GSH, CAT, and SOD were measured.

#### 2.3.5. Assessment of Oxidative Stress Biomarkers in Tissue Homogenate

##### Evaluation of the Enzymatic Activity of Catalase

CAT activity was determined spectrophotometrically according to the method described by Aebi [28]. The decomposition of hydrogen peroxide into water and oxygen by the CAT enzyme was observed using a spectrophotometer (240 nm, 1 min, 25 °C). Enzyme activity was determined as the unit of activity corresponding to nmol H_2_O_2_ destroyed/min/mL [29]. Measured 100 µL of tissue homogenate was added to 1.9 mL of phosphate buffer (50 mmol/L, pH 7.0) and 1 mL of freshly prepared H_2_O_2_ (2 mmol/L) and the mixture was transferred to the cuvette. The blank and standard were performed in the same manner, without tissue homogenate and with CAT instead of tissue homogenate, respectively.

The reading was taken spectrophotometrically at wavelength *λ* = 240 nm against the blank for 1 min. The results were expressed as units/mg of protein [28].

##### Evaluation of the Enzymatic Activity of Superoxide Dismutase

The evaluation of SOD is based on the reduction of nitro blue tetrazolium (NBT) to water-insoluble formazan blue; SOD inhibits the reduction of NBT, which can be measured at 560 nm by spectrophotometry [30]. A reaction mixture consisting of 200 µL of tissue homogenate, 1.2 mL of sodium pyrophosphate buffer (0.025 mol/L, pH 8.3), 100 µL of phenazine methosulfate (186 µmol/L), and 300 µL of NBT (300 µmol/L) was introduced into a test tube. The reaction was triggered by the addition of 200 µL of NADPH (780 µmol/L). After incubation at 30 °C for 90 s, a volume of 1 mL of glacial ACA was added to stop this reaction. Then, the reaction mixture was vigorously stirred with 4 mL n-butanol, allowed to stand for 10 min, and centrifuged. The butanol layer was separated and the intensity of the chromogen in the butanol layer was read at 560 nm against a blank. The calibration curve was performed with concentrations ranging from 10 to 100 µL of SOD as a standard. A linear regression equation was used to measure the activity of SOD [31].

##### Evaluation of the Enzymatic Activity of Reduced Glutathione

For the determination of GSH, the method described by Ellman was used [32]. In total, 50 μL of tissue homogenates were diluted in a phosphate buffer (10 mL, 0.1 M, pH 8). Then, 3 mL of the dilution mixture was transferred to cleaned test tubes, and 20 µL of DTNB (0.01 M) was added. The mixture was allowed to stand for the next 5 min, and then the absorbance was measured at a wavelength *λ* = 412 nm against a blank prepared under the same conditions without tissue homogenate [27,32].

##### Estimation of Lipid Peroxidation

The lipid peroxidation reflected by MDA levels was assessed using the method of Sastre et al. [33]. The MDA, the product of polyunsaturated fatty acid peroxidation in cells, is a good marker for the free radical induced damage and oxidative stress. The principle of this assay is based on the condensation of one molecule of MDA in a warm acidic medium (pH 2 to 3, 90–100 °C) with two molecules of TBA for a pink-colored chromogen that can be measured at 532 nm. A total of 500 μL of liver homogenate samples were added to 500 µL of 10% TCA acid and 1 mL of 0.67% TBA and centrifuged at 3000 rpm for 15 min. The collected supernatant was heated to 100 °C, in a water bath, for 15 min. After cooling, n-butanol was added to neutralize the mixture and the optical density of the samples was measured at 535 nm. The amount of MDA in the sample was expressed as mmol/g of tissue extract (liver and kidneys).

### 2.4. Statistical Analysis

All in vitro experiments were performed at least in triplicate. Analyses of all samples were run in triplicate and averaged. The data were statistically analyzed using one-way ANOVA followed by Dunnett’s test using GraphPad Prism 8.4.3 (GraphPad Software Inc., San Diego, CA, USA), and the values were expressed as mean ± SD. Pearson correlation analysis was used to evaluate the relationship between body and organ weight. A probability of *p* < 0.05 was considered as significant.

## 3. Results

### 3.1. Chemical Characterization of A. visnaga L. Essential Oil

The hydro distillation of *A. visnaga* L. plant gave a golden yellow essential oil, and the percentage yield was calculated to be 1.4% (*v*/*w* fresh material). The GC-MS analysis of the essential oil of *A. visnaga* L. resulted in a total of 33 compounds identified and eluted from between 1.195 and 23.902 min; β-ocimene, D-limonene, propanoic acid, 2-methyl-2-methylbutyl ester, linalool, pulegone, lavandulyl isobutyrate, and β-myrcene were the main compounds identified in the essential oil of *A. visnaga* L. with 86% comparison with the Wiley and NIST libraries, as it is presented in Table 1.

### 3.2. Total Phenol, Total Flavonoid, and Total Condensed Tannins Contents of A. visnaga L. Essential Oil

The total phenol contents (TPC) values in *A. visnaga* L. essential oil were determined based on the absorbance values, being expressed in terms of gallic acid equivalents (GAE) in reference to the standard curve equation:

*y* = 0.0133*x* − 0.01, *R*^2^ = 0.9902

The total flavonoid contents (TFC) were expressed in terms of quercetin equivalent using the standard curve equation:

*y* = 0.0175*x* − 0.0136, *R*^2^ = 0.9911

and the values ranged between 5.82 ± 0.79 g QE/g extract.

The total condensed tannin (TCT) content was 15.08 ± 1.76 catechin/g extract:

*y* = 0.0156*x* − 0.0184, *R*^2^ = 0.9902

TPC were in the range of 7.26 ± 1.68 extract (mg GAE/g extract), TFC ranged between 5.82 ± 0.79 (mg QE/g extract), and TCT ranged between 15.08 ± 1.76 (mg CE/g extract) (data are expressed as means ± standard deviation of triplicate samples).

### 3.3. Determination of the In Vitro Antioxidant Activities

The antioxidant potential of *A. visnaga* L. essential oil was evaluated using two different test assays: the free radical scavenging activity (DPPH) and the reducing power. Known antioxidant compounds, BHT and AA, were used as reference.

**Figure 1:**
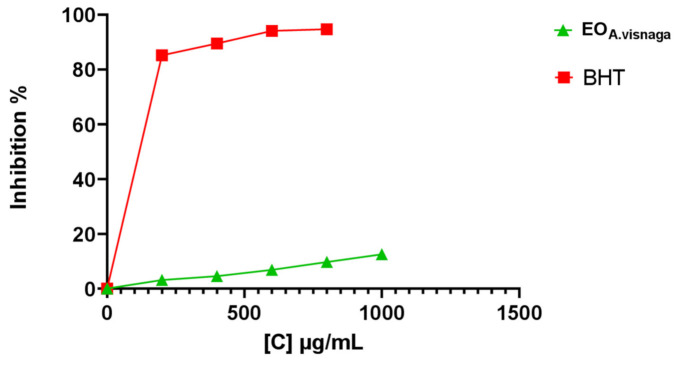
Percentage inhibition of the DPPH by different concentrations of essential oil of *A. visnaga* L. and standard (results expressed as means ± SD, of three parallel measurements (*p* < 0.05)). EO: essential oil; BHT: butylated hydroxytoluene.

The free radical scavenging capacities of the corresponding oil were measured by the DPPH test. According to the results obtained, the studied essential oil showed a low ability to reduce the DPPH free radical concentration with an IC_50_ value of 4.13 ± 0.22 mg/mL, in comparison with the reference antioxidant BHT (IC_50_ = 0.17 ± 0.01 mg/mL). The reduction in DPPH absorbance was dose-dependent. The inhibition rate of the DPPH-produced free radicals reached 12.53% at 1 mg/mL (Figure 1); this ability decreased with decreasing oil concentration, at 800 (9.68%), 600 (6.83%), 400 (4.55%), and 200 µL/mL (3.13%), respectively.

In the reducing power assay, the *A. visnaga* L. essential oil showed increased absorbance with the increase in the concentration, a dose-dependent effect similar to the AA standard (Table 2). In terms of the EC_50_ value, *A. visnaga* L. essential oil exhibited lower reducing power (0.730 ± 0.017 mg/mL) compared to that of the AA standard (EC_50_ = 0.034 ± 0.28 mg/mL) (values expressed are means ± SD, *n* = 3).

**Figure 2:**
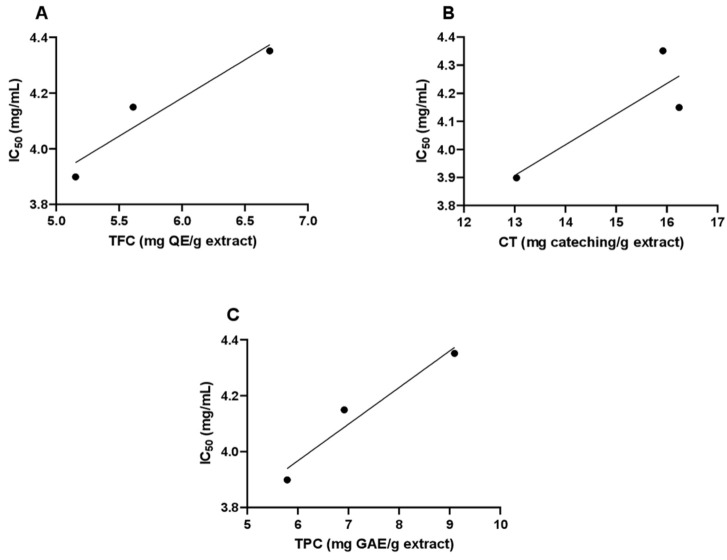
Pearson correlation graphics: (**A**) Pearson correlation between DPPH IC_50_ and flavonoids, (**B**) Pearson correlation between DPPH IC_50_ and Condensed Tannins, and (**C**) Pearson correlation between DPPH IC_50_ and phenols of *A. visnaga* L. TFC: total flavonoid content; CT: condensed tannins; TPC: total phenolic content; QE: quercetin equivalent.

Pearson correlation coefficients were calculated to identify the relationship between the antioxidant activity and the total phenolic, condensed tannins, and total flavonoid contents (Figure 2). The analysis revealed a positive correlation between DPPH/IC_50_ value and the total flavonoids, condensed tannins, and total phenolic content, with *r* = 0.958; *R*^2^ = 0.917, *r* = 0.851; *R*^2^ = 0.724, and *r* = 0.971; *R*^2^ = 0.942, respectively. A very strong positive correlation was observed for the phenols and flavonoids, suggesting that 94.2% and 91.7%, respectively, of the variability in antioxidant activity of *A. visnaga* essential oil is explained by the variability in TPC and TFC. The other 5.8 and 8.3% of variance is explained by unknown factors that were not measured in the experiment.

### 3.4. Determination of In Vivo Antioxidant Potential

#### 3.4.1. The Effects of the Essential Oil of *A. visnaga* L. on the Body Weight of the Treated Mice

The body weight of the mice was measured at the beginning (day 0) and at the end of the test (day 14); the results are presented in Table 3. For the control group, the AA group, and the two essential oil-treated groups (600 mg/kg and 1200 mg/kg, respectively), the body weight registered slight and normal increases throughout the treatment period without any statistical differences between day 0 and day 14 (*p* > 0.05). However, for the APAP group, there was a significant reduction in body weight at the end of the treatment (*p* < 0.05) compared to day 0.

The presence of body weight differences between treatment and control groups reported in Table 3 make the organ weight interpretation more complicated. To detect target organ damage, the relative organ weight to body weight was used (Table 4).

Based on the results in Table 4, a statistically significant decrease in the organ weights relative to body weight was observed in the kidneys and liver of mice exposed to APAP treatment (AA, APAP alone, AV1, and AV2) compared with the control group. In addition, a significant increase in the organ weights relative to body weight was recorded in the kidneys and liver of mice exposed to APAP (AA, AV1 and AV2) compared with the APAP toxic group (^#^ *p* < 0.05). These changes in the organ weights relative to body weight indicate a toxic effect of APAP on the animals’ organs (liver and kidneys) (*p* < 0.05).

Supplementation with AA and *A. visnaga* L. essential oil, except in mice models receiving an oral dose of AVI (i.e., 600 mg/kg PC of *A. visnaga* essential oil), showed a significant ability to thwart the toxic effect of APAP (^#^ *p* < 0.05).

Interestingly, a higher dose essential oil of *A. visnaga* (i.e., 1200 mg/kg PC) showed a comparable protective effect to the positive control AA in mice models (^#^ *p* < 0.05). The above observations indicate the hepato/renal protective activity of *A. visnaga* L. essential oil in a dose-dependent manner.

#### 3.4.2. Antioxidant Activity Assay

**Figure 3:**
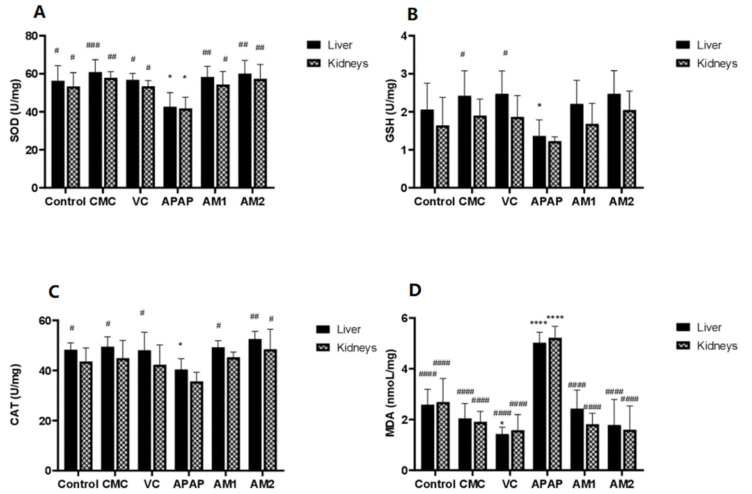
Effect of *A. visnaga* L. essential oil on antioxidant enzymes and MDA levels against acetaminophen-induced liver and kidneys injury in mice. Values are expressed as the mean ± SD (*n* = 5), * p < 0.05; **** p < 0.0001 vs. the normal control group and *^#^ p* < 0.05; *^##^ p* < 0.01; *^###^ p* < 0.001; *^####^ p* < 0.0001 vs. the toxic control group. (**A**) Effect of *A. visnaga* L. on the SOD level in APAP-treated mice liver and kidneys; (**B**) Effect of *A. visnaga* L. on the GSH level in APAP-treated mice liver and kidneys; (**C**) Effect of *A. visnaga* L. on the CAT level in APAP-treated mice liver and kidneys; (**D**) Effect of *A. visnaga* L. on the MDA level in APAP-treated mice liver and kidneys. SOD: superoxide dismutase; GSH: reduced glutathione; CAT: catalase; MDA: malondialdehyde; AA: ascorbic acid; CMC: sodium carboxymethyl cellulose; APAP: acetaminophen; AV1: essential oil of *A. visnaga* 600 mg/kg body weight; AV2: essential oil of *A. visnaga* 1200 mg/kg body weight.

The GSH, SOD, MDA, and CAT levels were measured in the liver and kidney tissues of the mice and are presented in Figure 3.

In the case of the liver homogenates, the toxic control group (III) showed significantly decreased levels of SOD, CAT, and GSH compared to the control, the standard AA, and the essential oil (600 and 1200 mg/kg body weight) -treated groups *(p* < 0.05). However, in the kidney homogenates, only SOD was significantly lower in the toxic group compared to the normal control (*p* < 0.05). The MDA content as a marker of lipid peroxidation, reflecting that the degree of oxidative stress was significantly higher in the groups receiving APAP in the homogenates of both tissues studied compared to the negative control and all other treated groups, including the essential oil-treated groups (*p* < 0.05).

Treatment with essential oil preserved the levels of SOD, CAT, and GSH at values comparable to the normal control (*p* > 0.05) and showed a significant dose-dependent decrease in the level of MDA when compared to the APAP toxic control in liver and kidney tissue (*p* < 0.05). The *A. visnaga* L. essential oil at 1200 mg/kg had the lowest MDA level compared to the normal and CMC control, but it was higher than the AA positive control. CAT activity did not show any significant differences between the groups.

## 4. Discussion

To date, very few studies exist on the *A. visnaga* L. essential oils and their antioxidant activity [34]. This study species showed a yield of essential oil of 1.4% *v*/*w* during the flowering period, higher than those mentioned by Khadhri et al. [35] and Khalfallah et al. [36] for Tunisian and Algerian species, respectively (yields ranging from 0.2–1.3%), or than those obtained during the fruiting period by Satrani et al. for the same Moroccan species [37] and by Günaydin et al. for a Turkish species (yields between 0.1–0.27%) [38]. The difference in yield of extracts can be influenced by factors such as origin, species, extraction methods, time of harvest, developmental stages, and geo climatic factors [39,40,41].

Chromatographic analysis showed that the main compound of the obtained essential oils were the terpenes. (Z)-β-ocimene was the main constituent, followed by the oxygenated monoterpenes D-limonene, linalool, pulegone and lavandulyl-butyrate. This composition differs from the one Günaydin et al. previously reported, characterized by nerol (29.98%), a-bisabolol (20.86%), and butylated hydroxytoluene (18.55%) [38]. While the Iranian *A. visnaga* L. essential oil was reported to be dominated by cis-pinene hydrate (42.83%), methyl octadecanoate (14.73%), and a-terpinene (9.20%), the composition of the essential oil resulted at the final of the present research was characterized by different monoterpenes as previously described by Sellami et al., with linalool (22.7–32%) as the main compound [35,42]. The presence of terpene alcohols such as the α-terpineol and of fatty acids, such as valeric acids, was mentioned by Feirouz and Salima [39]. The essential oils extracted from the North African *A. visnaga* L. plants (Algeria, Tunisia, and Morocco) show an analogy in their composition, but are dissimilar from those collected of the Iranian or Turkish species. These variations in chemical composition may be linked to ecological and genetic factors, such as soil type and proprieties, soil management, or environmental stress, which can lead to the modulation of certain enzymatic groups, responsible for the regulation of biosynthetic pathways [43,44,45].

The in vitro tests have shown that the essential oil of *A. visnaga* L. presents less antioxidant and phenolic activity than the reference agents. Our results revealed a lower or a relatively low content of total phenols, flavonoids, and condensed tannins (TPC = 7.26 ± 1.68 mg GAE/g extract; TFC = 5.82 ± 0.79 mg QE/g extract; CT = 15.08 ± 1.76 mg CE/g extract). El Karkouri et al. recently analyzed the same Moroccan species, and reported similar values to ours (of 7.16 mg GAE/g) for the crude extract obtained by Soxhlet extraction in methanol 70%, and lower values for the crude extract obtained by hydro-acetone, hydro-methanol, and Soxhlet extraction in ethanol [46]. Conversely, other studies analyzing the composition of the ethanolic and methanolic extracts of *A. visnaga* L. have reported higher TP, TF, and CT contents comparatively with our results, although this may partially be caused by the differences between extracts and essential oil [47,48].

The different concentrations of essential oil determined in this research showed low in vitro DPPH radical scavenging activity and low reducing power efficiency. These results are consistent with those of Aourabi et al. [47] and Keddad et al. [34], who reported an IC_50_ of 3.05 mg/mL at 1 mg/mL for the Moroccan *A. visnaga* aqueous extract and a 9.16% DPPH inhibition at 1 mg/mL for the Algerian *A. visnaga* L. In contrast to these results, Darkaoui et al. reported for the Moroccan species an IC_50_ of 56.053 ± 0.856 µg/mL [49], lower than that obtained in our study, while the antioxidant activity of the methanolic extract of the Palestinian *A. visnaga* L. showed better results (IC_50_ = 6.07 ± 2.14 μg/mL) with an inhibition percentage of 90.63% at 100 µg/mL [50].

Concerning the in vitro reducing power, essential oil showed good activity at an EC_50_ = 730.53 ± 0.017 µg/mL, although this is relatively lower when compared to other species within the *Apiaceae* family, such as the hydroalcoholic extract of the aerial parts of *Ammoides atlantica* with an IC_50_: 92.70 ± 1.00 µg/mL, the Tunisian *Pimpinella anisum* during maturation (EC_50_ = 0.523 mg/mL), or the ethanolic seed extract of *Trachyspermum ammi*, with a reducing power ability at an optic density of 0.91 at 25 μg/mL [51,52,53].

The slightly lower antiradical activity of our sample can be explained by the deficiency in phenolic content, which is suggested by their non-dominance in the chemical composition. In this regard, strong correlations were observed between the antioxidant activity, TPC, TFC, and CT at a 95% confidence level. These results agree with the reports of the literature that show that the bleaching of the DPPH solution is strongly correlated with the amount of total phenolic content [54,55,56].

To our knowledge, this is the first study to evaluate the antioxidant potential of *A. visnaga* L. essential oil in vivo. The results of this study revealed that APAP treatment induced oxidative stress in the liver and kidney of mice, which was characterized by increased levels of MDA, a marker of lipid peroxidation, as well as reductions in the antioxidant activities SOD, CAT, and GSH. An upregulation of lipid peroxidation is reported to induce subcellular and tissue disruption in liver and kidney [57,58]. Other studies have reported APAP-induced oxidative stress, with massive lipid peroxides production and decreased levels of SOD, CAT, and GPx [59,60,61]. The decrease in the antioxidant defense status of SOD, CAT, and GSH accompanied by the increase in LPO in liver and kidney tissues after APAP exposure could be attributed to ROS excessive generation and subsequent cellular damage of the healthy cells capable of halting oxidative attack [62,63]. The endogenous detoxification provided by the lowered SOD, CAT, and GSH activities would, thus, be insufficient in scavenging the superoxide anions and hydroxyl ions [1,64].

The administration of *A. visnaga* L. essential oil against APAP resulted in an increase in SOD, CAT, and GSH activities and a statistical decrease in MDA levels in mouse liver and kidney tissues. In this context, we have found an inconsistency between the in vivo very good antioxidant efficiency and the in vitro low-moderate radical scavenging activity. This interesting in vivo/in vitro difference has been reported elsewhere for other antioxidants, such as inulin [65], berry juices [66], or milk-derived antioxidative peptides [67], where the structure of the compound imprints on each of the substances a unique behavior [68]. The potential synergy with other compounds along the metabolic pathways could be one cause to trigger the significant increases in the antioxidant activity, less predictable by the indirect in vitro assays executed under specific conditions [67]. However, as Slatnar et al. suggested, these differences in antioxidant capacity could rest in the molecular nature of the bioactive compounds [64]. Pietta et al. reported, similarly to us, ineffective in vitro antioxidant activity but important antioxidant in vivo effects in the case of a ginkgo extract with standardized content of polyphenols [69]. However, not the polyphenols, but the terpenic ginkgolides present in the ginkgo extracts and insensitive to the used in vitro assay were later found to play a major antioxidant role in vivo [69,70]. In our case, the antioxidant activity may also derive, in large part, from the specific terpene composition that was identified by GC-MS analysis: the monoterpenes (Z)-β-ocimene, linalool, pulegone, and lavandulyl butyrate.

Significant antioxidant properties of monoterpenic compounds were determined previously in the essential oil extracted from leaves and fruit of *Schinus molle* L. [64]. Similar antioxidant activity was reported by Ruberto et al., for the monoterpenes α-pinene, β-pinene, limonene, β-myrcene, sabinene, and terpinolene [71]. Both α- and β-terpinolene exhibit antioxidant activity comparable to that of α-tocopherol [70,72]. Moreover, Edziri et al. reported that the high percentage of monoterpenes identified in the essential oils of *Retama raetam* (Forssk.) Webb (Fabaceae) shows good antioxidant activity [73]. In line with our results, ocimene and linalool measured by a Total TRAP assay and piperitenone from *Mentha longifolia* L. essential oil exhibit a high antioxidant potential [74,75]. Administration of linalool significantly reduces liver MDA excess levels in mice treated with lipopolysaccharide (LPS)/D-galactosamine by downregulating myeloperoxidase activity [76]. The pulegone is also reported for its significant role in lipid peroxidation decrease (MDA marker) coupled with increasing antioxidant levels [76].

The present study clearly demonstrates that intraperitoneal injection of APAP at a dose of 400 mg/kg body weight significantly decreased the body weight of mice and the relative organ weight at the end of the experiment. In this context, Abdul Hamid et al. also observed that treatment of male Sprague-Dawley rats with APAP decreased body weight and kidney weight [77]. In the same way, Jaeschke et al. have indicated that APAP overdose is strongly associated with hepatotoxicity and nephrotoxicity in humans and animal models [78]. In humans, when the glucuronidation and sulfation pathways are saturated, the 10% of APAP that is not metabolized and excreted in the urine is oxidized by cytochrome P450 2E1 to form the toxic intermediate N-acetyl-p-benzoquinone imine (NAPQI) [79]. At therapeutic doses, this unstable metabolite is detoxified by conjugation to glutathione (GSH) and eliminated via the urine or bile [79]. Upon APAP overdose, overproduced NAPQI results in GSH depletion and binds to proteins to form an APAP–protein adduct, thereby inducing cellular oxidative stress at the hepatic level [79]. A similar mechanism was observed in renal tissue, in male Wistar albino rats: an overdose of APAP estimated by 900 mg/kg body weight induced nephrotoxicity, which was reflected by a significant increase in renal MDA, in addition to significant reductions in renal reduced glutathione (GSH) [80]. Supplementation with *A. visnaga* L. essential oil provided protection against APAP-induced hepatotoxicity and nephrotoxicity and restored relative organ weights in mice in a dose-dependent manner, through downregulation of MDA levels and upregulation of GSH and other antioxidant enzymes.

With regard to the risk of toxicity by administration of the essential oil, it is reported in previous toxicity studies on *A. visnaga* L. seeds that neither ethanolic extract nor aqueous extract reveal any signs or symptoms of toxicity in rats at a limit dose of 5000 mg/kg [81,82]. To obtain the human equivalent dose (HED) according to the FDA guidelines [83], it was calculated as follows:

HED = animal dose × (Animal Km/Human Km)

where Km is the factor for converting mg/kg dose to mg/m^2^ dose, considering an average human body weight at 60 kg (Km = 37) and an average mouse weight at 20 g (Km = 3). This resulted in a HED value of 0.097 g/kg for our highest dose of 1200 mg/kg, which is too low to provoke toxicity leading to mortality, according to the LD50 values reported in the literature (toxic doses at 60 g for an adult weighing 60 kg) [84].

Regarding the absorption and metabolism of *A. visnaga* essential oil, its major compounds, including limonene and linalool, were reported to have good dermal, pulmonary, and oral absorption [85,86,87]. Due to its lipophilic nature, the limonene is quickly and easily diffused and absorbed by the skin, with a rapid increase in plasma concentration at 10 min after dermal application in both humans and mice models [86,87]. In humans, repeated exposure to limonene in inhalation chambers, at 10, 225, and 450 mg/m^3^, resulted in a 70% absorption rate into the blood from the pulmonary alveoli. A blood clearance of 1.1 l/kg/h was observed after 4 h, indicating easy metabolism of limonene by the body [88,89]. Linalool is also reported to have an up to 90% absorption rate at the gut level in only 7 h after oral administration in rats, at a dose of 500 mg/kg [89]. In addition, the quantification of human whole blood showed the appearance of linalool 25 min after inhalation and decreasing around 40–45 min. In total, 58% of the ingested dose of linalool was eliminated and excreted in urine, 25% as ^14^CO_2_ in exhaled air, and 16% in faces after 72 h, via the induction of cytochrome P450-dependent monooxygenase and liver microsomal uridine diphosphate glucuronyl transferase [90]. This suggests that linalool metabolites were able to enter intermediate metabolic pathways without risk of long-term exposure due to tissue accumulation [90].

At present, very few studies have provided evidence regarding the safety or efficacy of this essential oil through clinical trials. Translational studies are necessary to predict the metabolism and potential toxicity, as well as systematic clinical trials to test the action of *A. visnaga* L. essential oil in targeted or generalized therapies.

## 5. Conclusions

The present study examined the chemical composition and antioxidant activities in vitro and in vivo of *A. visnaga* L. plant. GC-MS analysis of the essential oil of *A. visnaga* L. showed a strong presence of terpenoids, with Z-(β)-ocimene and pulegone as two major components. In vitro, the natural essential oil expressed low DPPH radical scavenging activity and reduced antioxidant power; this is due to the low phenolic composition. The in vivo study on Swiss albino mice showed that dietary supplementation of *A. visnaga* L. essential oil significantly improved the antioxidant status marked by an increase in the antioxidant enzymatic activities of SOD, CAT, and GSH-Px, and the decrease in the concentration of MDA. Based on the above results, the high antioxidant activity observed in vivo may be due to the richness of *A. visnaga* L. essential oil in terpene compounds, which may be useful in the treatment of oxidative stress related diseases.

## Figures and Tables

**Table 1:** Chemical composition of *A. visnaga* L. essential oil.

Compounds	^a^ IR	^b^ IR	Area %
Propanoic acid, 2-methyl-, butyl ester	1011	944	3.40
Bicyclo[3.1.0]hexane, 4-methylene-1-(1-methylethyl)-	1018	987	1.23
β-myrcene	1022	1003	1.36
Butanoic acid, 2-methyl-, 2-methyl propyl ester	1025	1015	4.39
Butane, 1-(ethenyloxy)-3-methyl-	1030	-	12.28
4-Carene	1034	1030	1.61
(Z)-β-ocimene	1037	1037	4.03
Butanoic acid, 2-methyl-, pentyl ester	1059	1121	16.13
Linalool	1064	1097	22.94
D-Limonene	1086	1030	0.69
Pulegone	1209	1209	5.45
Lavandulyl isobutyrate	1275	1404	0.48

^a^ RI (retention index) measured relative to n-alkanes (C6–C30) on the non-polar 123 DB11 column. ^b^ Linear retention index taken from the NIST 05 library and the literature.

**Table 2:** Reducing power of *A. visnaga* L. essential oil at different concentrations.

Sample	Sample Concentration (µg/mL)				
	200	400	600	800	1000
Essential oil	0.13 ± 0.01 *	0.29 ± 0.02 *	0.38 ± 0.04 *	0.57 ± 0.005 *	0.64 ± 0.005 *
Ascorbic acid	0.43 ± 0.01	0.71 ± 0.01	0.95 ± 0.03	1.21 ± 0.01	1.52 ± 0.005

Values expressed are means ± SD, *n* = 3, * *p* < 0.05.

**Table 3:** Body weight of the mice during the treatment period.

Groups	Mean Body Weight in Gram ± SD	
	Day 0	Day 14
C	29.39 ± 0.29	29.58 ± 0.24
CMC	30.48 ± 0.31	30.71 ± 0.30
APAP	32.54 ± 0.43	29.78 ± 0.65 *
AA	27.47 ± 0.28	27.92 ± 0.72
AV 1	34.53 ± 0.29	35.09 ± 0.25
AV 2	25.71 ± 0.39	26.42 ± 0.88

C: Normal control; CMC: vehicle group–carboxymethyl cellulose 0.1%; APAP: Toxic control treated with acetaminophen 400 mg/kg body weight (ip); AV1: essential oil of *A. visnaga* 600 mg/kg body weight; AV2: essential oil of *A. visnaga* 1200 mg/kg body weight; AA: Ascorbic acid 200 mg/kg body weight. All data are mean ± S.D (*n* = 5/group), * *p* < 0.05 APAP at Day 0 vs. APAP at Day 14.

**Table 4:** Organ weights relative to body weight of Swiss Albinos mice.

Organs	Groups					
	C	CMC	AA	APAP	AV1	AV2
Kidneys	1.37 ± 0.11	1.36 ± 0.17	1.24 ± 0.08 *;#	1.04 ± 0.14 **	1.15 ± 0.10 *	1.19 ± 0.07 *;#
Liver	5.26 ± 0.26	5.05 ± 0.11	4.66 ± 0.16 **;###	3.88 ± 0.13 ***	4.04 ± 0.18 ***	4.83 ± 0.35 *;##

All values are expressed as mean ± SD. C: Normal control; CMC: vehicle group–carboxymethyl cellulose 0.1%; APAP: Toxic control treated with acetaminophen 400 mg/kg body weight (ip); AV1: essential oil of *A. visnaga* 600 mg/kg body weight; AV2: essential oil of *A. visnaga* 1200 mg/kg body weight; AA: Ascorbic acid 200 mg/kg body weight (significant differences as compared with the normal control group * *p* < 0.05; ** *p* < 0.01; *** *p* < 0.001; significant differences as compared with the toxic control group ^#^ *p* < 0.05; ^##^ *p* < 0.01; ^###^ *p* < 0.001).

## Data Availability

Data is contained within this manuscript.
